# Does colour variety accurately quantify nutritional value in children's lunchboxes? A pilot study

**DOI:** 10.1016/j.puhip.2023.100363

**Published:** 2023-01-31

**Authors:** D.E. Courtney, É.J. O'Reilly

**Affiliations:** School of Public Health, University College Cork, 4th Floor, Western Gateway Building, Western Road, Cork, Ireland

**Keywords:** Colour, Eating, Children, Fruit, Vegetables, Public health nutrition

## Abstract

**Objectives:**

In advocating a diet rich in fruit and vegetables, public health authorities emphasise dietary colour variety to ensure adequate nutrient consumption. The relationship between food colour variety and nutritional value in children's lunches attending one school was evaluated.

**Study design:**

Observational cross-sectional study.

**Methods:**

Eighty-two children had their lunchboxes photographed over one day. Foods were scored using the Nutri-score labelling system, assigning foods a letter based on nutritional content. Composite Nutri-scores were calculated as average total Nutri-score per food per lunchbox. Eleven observers used a colour chart to assign colours to foods from a sample of lunchboxes; intra-class correlation was calculated. Associations between nutrient scores and colour variety were estimated.

**Results:**

Lunchboxes contained an average of 4 ± 1 colours. Number of colours did not vary by age or sex. Two thirds of lunches had composite Nutri-scores of C or better, with better scores in older children. There was no association between colour variety and composite Nutri-score. Fruit, vegetables, whole grains and fats were associated with greater colour variety. Vegetables and meat, fish and eggs were associated with better scores; refined grains, dairy, snacks, fats and condiments were associated with worse.

**Conclusions:**

Although this study did not demonstrate a relationship between colour variety and nutritional score, findings suggest that lunches containing fruit and vegetables tended towards greater colour variety than those without. This indicates that adjusting guidelines to incorporate food colour variety may be of use in future iterations of dietary guidelines for children.

## 1 Introduction

Childhood obesity is one of the most serious public health challenges of the 21st century with prevalence among those aged between 5 and 19 years rising from 1% to 7% between 1975 and 2016 [1]. Some of these children also face the double burden of under-nutrition [2,3].

Promoting the consumption of fruit and vegetables, abdundant in colour, is one suggested approach to eliminating obesity and improve nutrient intake [4]. Nutritional agencies endorse a colourful diet to maintain good health [[5], [6], [7]], while successful, sustained weight loss has been shown to be associated with higher consumption of colourful foods, such as fruit and vegetables [[8], [9], [10]].

König et al. found that colour variety may present an intuitive cue for nutritious food choices [[11], [12], [13]]. They observed that more colourful meals were associated with healthier eating patterns due to higher fruit and vegetable consumption and lower intake of processed snacks [11,12]. Conversely, less colourful meals contained more sugary foods [13].

Children attending Irish primary schools consume three servings of fruit and vegetables daily, substantially below the recommended 5 to 7 [14]. Additionally, homemade lunches tend to be lower in whole grains and higher in fat and sugar [15]. This study explores colour variety in children's lunchboxes to ascertain whether more colour equates to healthier. The aim of the study is to inform healthy eating guidelines through offering an easy-to-follow alternative to more complex recommendations.

## 2 Methods

### 2.1 Study design and population

This study was observational in design, providing a snapshot of childrens’ lunchboxes in a single school. Increasing its scope was not possible due to the COVID-19 pandemic.

### 2.2 Study population and sampling

Due to COVID-19 restrictions, the study was amended to a single-school pilot with convenience sampling. The primary researcher (DEC) established a relationship with an urban school in the north-midlands of the Republic of Ireland. This school, a member of Green Schools Ireland [16], has a history of engagement with healthy eating initiatives [17] and a school lunch policy. Despite COVID-19 restrictions imposed, the school principal was keen to progress the research.

Eligible participants were identified as students from years 5–8. Children in these years have a longer school day allowing more time to photograph lunchboxes while complying with time-consuming COVID-19 measures. There were no exclusion criteria. Ninety-eight participants were eligible for recruitment. In this school, no food was provided to children (as is the case in 50% of Irish primary schools [18]); all lunches were brought from home. In total, 87 children consented to partake in the study. On the day of photographing, 5 were absent, resulting in a total of 82 participants.

### 2.3 Data collection

Prior to study commencement, the principal was coached on UCC's data collection policies and procedures. Data were gathered in line with COVID-19 public health measures. Information leaflets and consent forms were distributed to children and their parents/guardians one month prior to data collection (Supplementary Figs. 1 and 2). A delay between consenting and the study day was to reduce information bias following recruitment. Four weeks later, images of participants' lunchboxes were captured. Each was assigned an identifier number. Children's age, sex and year were noted and anonymised. Other variables, including weight and height, included as part of the initial protocol, were later excluded to facilitate maximum participation and because the researcher was not able to attend school due to COVID-19 restrictions.

Data collection and processing included documenting individual foods, scoring foods with the Nutri-score calculator, quantifying number and degree of colour variety per lunchbox and calculating whether correlations exist between number of colours and composite Nutri-score. Subjective data involved independent reviewers assigning colours to foods.

### 2.4 Quantification of food items

Foods were identified and documented from photographs. Lunchboxes contained combinations of 92 foods. Each food was grouped into one of nine categories; fruit, vegetables (including légumes), whole grains, refined grains, meat, fish and eggs (MFE), nuts, dairy, snack foods and fats and oils (Supplementary Table 3). Condiments, such as strawberry jam and tomato sauce, were separated. Fats and oils comprised only indeterminate spreadable vegetable oils/butter. However, because face-to-face and other contact with parents and children was not permissable at the time of data collection, we relied on a 2018 survey of nationally representative Irish residents that found that full-fat butter is the most common spread used in Irish households. Therefore, this item was coded as butter [19]. The only mixed foods observed were store-bought smoothies; no soups were included.

### 2.5 Measure of nutritional value

Nutritional information including energy (kJ), total fats (g), saturated fats (g), sugar (g), protein (g), fibre (g) and salt (g) per 100g were input into the Nutri-Net Santé calculator [20]. In accordance with website recommendations, cheeses, beverages and fats were classified separately. Adjusted calculators consider protein indicative of calcium content in the case of cheese, distinguish between animal and plant fat and allow for consistency across beverages [21]. The Nutri-Scores of nuts were calculated according to work done by Braesco et al., with adjustments made discounting saturated fatty acid content and energy density; this has been shown to improve the score's precision [22]. As some foods, including breads and spreads, were not packaged in their original packaging, nutritional information, based on market share of Irish grocery stores, was reviewed to ensure accurate spread of brands across all major supermarkets [23]. Foods were randomly assigned to supermarkets based on market share. Nutritional information on 22% of included foods were from Supervalu, 22% from Tesco, 21% from Dunnes, 13% from Lidl, 12% from Aldi and the remainder from ‘Others’, including Eurospar and Fresh. Nutritional information on other foods without front-of-pack labels, such as fruit, was gathered from nutrient analyses by the Department of Health in the United Kingdom [24] and the USDA National Nutrient Database [25].

The Nutri-Score of each food was calculated and assigned a score from A (‘very good’) to E (‘bad’); a Nutri-score of A was assigned a numerical value of 1 and so on, through to E which was assigned a numerical value of 5. The sum of the total score per lunchbox was computed. Composite Nutri-score was calculated by dividing the total score by the number of items per lunchbox, producing an average numerical value between 1 and 5.

The image below provides an example lunchbox containing a red apple, mandarin, brown sliced pan, ham and butter. Nutritional content of the apple and mandarin were extracted from the UK database, while that of sliced pan was of Dunnes homebrand, with Supervalu-purchased crumbed ham and butter from Tesco. Utilising the Nutri-score calculator yielded Nutri-scores of A, B, A, C and E, respectively, resulting in a total numerical score of 12 (1 + 2 + 1 + 3 + 5). Five colours - red, orange, brown, pink and yellow, respectively - were documented. Total numerical score was divided by the number of foods to give a score of 2.4, equating to a Nutri-score of B or ‘good’ nutritional value. This process was repeated for each lunchbox.

**Figure:**
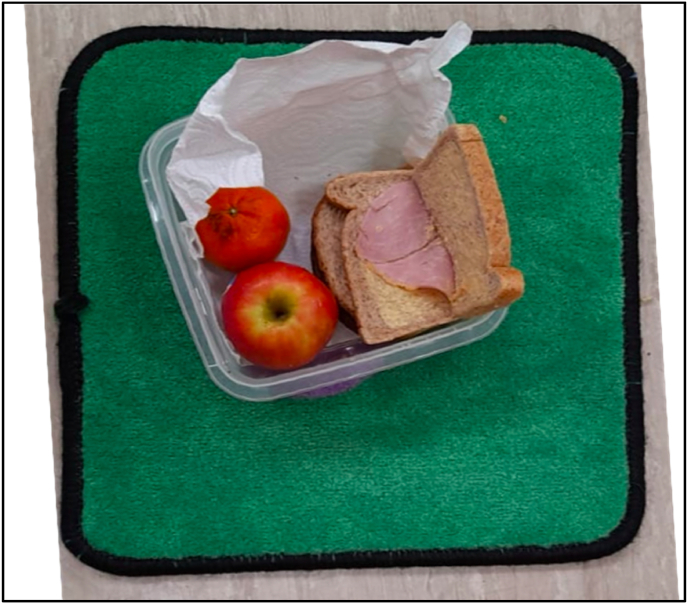

### 2.6 Colour perception

A sample of lunchboxes was presented to eleven independent observers who rated the colour of each food based on a 13-colour chart created by DEC. Observers were asked to choose the most appropriate colour to describe each food. The most popular colour was selected based on observer consensus.

The Strengthening the Reporting of Observational Studies in Epidemiology (STROBE) checklist [26] was applied and presented (Supplementary Fig. 4).

### 2.7 Statistical analysis

Data were analysed with IBM SPSS (Version 26) using descriptive and inferential statistics, including Spearman's rank-order correlation, Mann Whitney U testing, Fisher's exact testing, chi-square tests for association and intra-class correlation (ICC).

### 2.8 Ethical considerations

Ethical approval was sought and approved by University College Cork School of Public Health Social Research Ethics Committee in March 2021. Participants, their parents and school principal provided written consent prior to participation.

## 3 Results

### 3.1 Demographics, nutri-score and food groups

Composite Nutri-score per lunchbox was 3.0 ± 1 (mean ± standard deviation (SD)), equating to a Nutri-score of C or ‘average’ nutritional quality. Of 82 lunchboxes, 2% (*n* = 2) had a composite Nutri-score of A, 28% (*n* = 23) B, 35% (*n* = 29) C, 26% (*n* = 21) D and 8.5% (*n* = 7) E. Median age was 10 years with a mean of 10.3 ± 1.1 (mean ± SD) years. Half were female. Participants were distributed evenly across all school years. The most common food group was fruit (*n* = 73, 89%) followed by refined grains (*n* = 64, 78%) while 5% (*n* = 4) had nuts (Table 1).

**Table 1:** Demographics, composite Nutri-score and number of colours in children from pilot school who consented to have their lunchboxes photographed (*n* = 82).

	Frequency (*n*)	Percentage (%)
Sex		
Male	41	50
Female	41	50
Age		
9 years	26	32
10 years	21	26
11 years	18	22
12 years	17	21
Class breakdown		
Year 5	16	20
Year 6	17	20
Year 7	22	27
Year 8	27	33
Composite Nutri-score per lunchbox		
A	2	2
B	23	28
C	29	35
D	21	26
E	7	9
Number of colours per lunchbox		
3 or less	15	18
4	39	48
5	21	26
6 or more	7	9
Most commonly observed colours		
White	65	79
Yellow	53	65
Red	51	62
Golden	43	52
Orange	35	43
Pink	33	40
Green	27	33
Food groups (in order of frequency)		
Fruit	73	89
Refined grains	64	78
Dairy products	51	62
Snack foods	38	46
Meat, fish & eggs	32	39
Fats & oils	22	27
Whole grain	17	21
Vegetables (including légumes)	13	16
Condiments	11	13
Nuts	4	5

### 3.2 Colour variety

Lunchboxes contained a mean of 4.2 ± 1 (mean ± SD) colours. Two participants (2.4%) had 2 colours or fewer while 28 (34%) had 5 or more. The most popular colour was *white* (*n* = 65, 79%), almost two thirds (*n* = 53) had lunches containing *yellow* while more than half (*n* = 51) had *red* (Supplementary Tables 5(a) and 5(b)).

Results of tests of association are presented in Table 2, Table 3, Table 4, Table 5, and Supplementary tables and Fig. 6 through 8. Spearman's rank-order correlation found no association between number of colours per lunchbox and age (*r*_*s*_ = −0.159, *p* = 0.154). Mann Whitney U testing showed no difference (*U* = 730.50, *p* = 0.274) between number of colours per lunchbox and sex. Fisher's exact testing demonstrated a correlation between the colour *golden* and age (2-tailed Fisher's exact test, *p* = 0.043), with younger children having more golden foods than older. Chi-square testing found a relationship between the colour *white* and sex (*p* = 0.005), with girls more likely than boys to have *white* foods. There were significant relationships between colour variety and fruit (*p* = 0.007), vegetables (*p* < 0.001), whole grains (*p* = 0.020), MFE (*p* = 0.026) and fats and oils (*p* = 0.038); this corresponds with our expectations that increased colour could serve as a proxy for higher nutritional value.

**Table 2:** Tests of association between number of colours per lunchbox, composite Nutri-score per lunchbox and child's age.

	Variables		Number of colours	Composite Nutri-score
Spearman's rho^a^	Number of colours per lunchbox	Correlation coefficient	1.000	−.159
		Sig. (2-tailed)		.154
		*N*	82	82
	Age	Correlation coefficient	−.159	1.000
		Sig. (2-tailed)	.154	
		*N*	82	82
	Composite Nutri-score	Correlation coefficient	1.000	−.266
		Sig. (2-tailed)		0.016**
		*N*	82	82
	Age	Correlation coefficient	−.266	1.000
		Sig. (2-tailed)	.016**	
		*N*	82	82
	Number of colours per lunchbox	Correlation coefficient	1.000	−.090
		Sig. (2-tailed)		.421
		*N*	82	82
	Composite Nutri-score	Correlation coefficient	−.090	1.000
		Sig. (2-tailed)	.421	
		*N*	82	82

*Statistically-significant associations (p < 0.05) are emphasised in boldface.

a Spearman's Rank-Order Correlation.

**Table 3:** Mann Whitney *U* Test presenting the correlation between number of colours per lunchbox or composite Nutri-score per lunchbox and child's sex.

Variables	Sex	*n*	Median (range)	Mean Rank	Test Statistic^a^	Asymp. Sig. (2-tailed)
Number of colours per lunchbox	Female	41	4 (4)	38.82	730.50	.274
	Male	41	4 (5)	44.18		
Composite Nutri-score	Female	41	3 (4)	39.21	746.50	.363
	Male	41	3 (4)	43.79		

a Grouping variable: Sex.

**Table 4:**
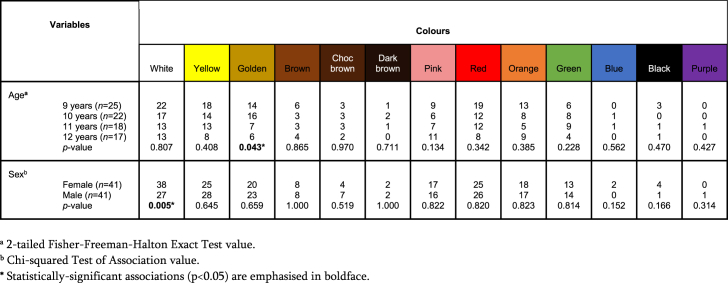
Number of times individual colours were observed in lunchboxes (*n* = 82) stratified by age and sex.

**Table 5:** Mann Whitney *U* Test presenting the correlation between number of colours or composite Nutri-score and food group.

Variables	Food group	*N*	Mean	Test Statistic	Asymp. Sig. (2-sided test)
Number of colours	Fruit	71	4.34	207.00	.007*
	No fruit	11	3.45		
	Vegetables	17	4.94	259.00	<.001*
	No vegetables	65	4.03		
	Whole grains	17	4.59	363.00	.020*
	No whole grains	65	4.12		
	Refined grains	64	4.19	501.00	.368
	No refined grains	18	4.33		
	Meat, fish & eggs	32	4.50	581.50	.026*
	No meat, fish & eggs	50	4.04		
	Nuts	4	3.25	96.00	.209
	No nuts	78	4.27		
	Dairy	51	4.29	693.5	.320
	No dairy	31	4.10		
	Snacks	40	4.37	692.00	.141
	No snacks	42	4.07		
	Fats & oils	22	4.59	475.00	.038*
	No fats & oils	60	4.08		
	Condiments	11	4.09	340.50	.466
	No condiments	71	4.24		
Composite Nutri-score	Fruit	71	3.01	256.5	.056
	No fruit	11	3.64		
	Vegetables	17	2.65	369.50	.029*
	No vegetables	65	3.22		
	Whole grains	17	2.71	395.50	.061
	No whole grains	65	3.20		
	Refined grains	64	3.27	326.50	.004*
	No refined grains	18	2.50		
	Meat, fish & eggs	32	2.66	462.50	<.001*
	No meat, fish & eggs	50	3.38		
	Nuts	4	3.06	146.50	.83
	No nuts	78	1.95		
	Dairy	51	3.31	545.00	.014*
	No dairy	31	2.74		
	Snacks	40	3.43	524.50	.002*
	No snacks	42	2.79		
	Fats & oils	22	3.64	389.00	.003*
	No fats & oils	60	2.90		
	Condiments	11	3.64	244.00	.037*
	No condiments	71	3.01		

* Statistically-significant associations (p < 0.05) are emphasised in boldface.

### 3.3 Nutri-score

Spearman's rank-order correlation found a relationship between composite Nutri-score and age (*r*_*s*_ = −0.266, *p* = 0.016), with older children having better scores versus younger. Mann Whitney U testing found no association between composite Nutri-score and sex (*U* = 746.50, *p* = 0.363). Mann Whitney U testing found relationships between composite Nutri-score and vegetables (*U* = 369.50, *p* = 0.029), refined grains (*U* = 326.50, *p* = 0.004), MFE (*U* = 462.50, *p* < 0.001), dairy products (*U* = 545.00, *p* = 0.014), snacks (*U* = 524.50, *p* = 0.002), fats and oils (*U* = 389.00, *p* = 0.003) and condiments (*U* = 244.00, *p* = 0.037). These results provide a mixed picture of the value of composite Nutri-score calculations in this group.

### 3.4 Correlations between nutri-score and colour variety

Spearman's *rho* found no association between number of colours and composite Nutri-score (*r*_*s*_ = −0.090, *p* = 0.421).

### 3.5 Inter-rater reliability

Inter-rater reliability (IRR) determined the degree of agreement between observers. IRR was calculated using ICC with values of less than 0.5 indicating poor reliability, values between 0.5 and 0.75 indicating good reliability and values between 0.75 and 0.9 indicating excellent reliability. IRR was greater than 0.9 indicating minimal variability between observers (Supplementary Table 8).

### 3.6 Assessment of study quality

Study feasibility fulfilled all criteria required for observational studies according to the STROBE checklist (Supplementary Fig. 4). Acceptability was high with recruitment and refusal rates of 84.5% and 5.4%, respectively. While parental interest was measured virtually, through email contact, and was minimal (*n* = 2), the questions posed, one regarding study preparation and the other requesting information on results, were viewed as positive indicators of acceptability.

## 4 Discussion

This is the first pilot of its kind endeavouring to create a template for more easily making healthy food choices, using colour variety as a proxy for nutritional content. While the study does not provide evidence of the association between colour variety and overall nutritional score, it did find that fruit, vegetables, whole grains and fats were associated with greater colour variety.

The Nutri-score comprises a five-colour front-of-pack labelling system evaluated with the Food Standards Agency nutrient profiling system (FSAm-NPS) [[27], [28], [29]]. It has been shown to be robust in characterising foods' nutritional content and encapsulating the nutritional quality of diets through individualised scores. One German study compared the Nutri-score to German Food-Based Dietary Guidelines across 8587 foods and found that 80% of fruits and vegetables were classified, as expected, as A (‘very good’) or B (‘good’) while 93% of sugary snacks were classified as D (‘poor’) or E (‘bad’), showing good discrimination and consistency [30]. Similar results were found in a comparative study of 8 European countries when compared to European Food Information Resource (EUROFIR) databases [31]. Due to these consistencies [[31], [32], [33]], the Nutri-score has been validated for utilisation in many countries [21,30,33].

One third of children in this group had lunches with a composite Nutri-score of D or worse, corresponding with ‘poor’ or ‘bad’ nutritional value. Almost 80% had lunches containing refined grains, such as white bread and crackers. Most children (89%) had lunches containing fruit but fewer than 1 in 5 had vegetables, vegetables having the highest composite Nutri-score of all groups. *White* was the most commonly observed colour, of which 59% comprised refined grains, 27% dairy products, 10% snack foods and 4% MFE. The colour *golden* correlated significantly with age, with younger children more likely than older to have *golden* foods in their lunchboxes; this may be attributable to preferences surrounding colourful packaging accompanying snack foods and warrants investigation. *White* foods were more prevalent in girls' lunchboxes versus boys, perhaps relating to the sample comprising relatively pickier eaters who prefer the media-dubbed ‘whiteatarian diet’. [34]. Those with fruit had a mean of 4.3 colours in their lunches versus 3.4 in those without (Mann Whitney *U* = 207.00, *p* = 0.007) while those with vegetables had a mean of 4.9 colours versus 4.0 in those without (Mann Whitney *U* = 259, *p* < 0.001). The suggestion is that fruit and vegetables, with respective composite Nutri-scores of B and A, lead to more colourful, healthier lunches. These findings were countered by a correlation between fats and number of colours where lunches containing fat had a mean of 4.5 colours compared to 4 in those without (Mann Whitney *U* = 475.00, *p* = 0.038). Lunches containing MFE had greater colour variety than those that did not, with 4.5 colours in those with MFE versus 4 in those without (Mann Whitney *U* = 581.50, *p* = 0.026).

There was no association between number of colours and composite Nutri-score; this is unsurprising in an inadequately-powered study. Number of colours was neither associated with age nor sex. Composite Nutri-score was associated with age; poorer Nutri-scores were observed in younger versus older children (Spearman's rank order correlation, *r*_*s*_ = −0.266, *n* = 82, *p* = 0.016) indicating that older children tended towards having relatively more nourishing foods. Composite Nutri-score was associated with fats; those with butter had a composite Nutri-score of D versus C in those without (Mann Whitney *U* = 389.00, *p* = 0.003). Similarly, there was an association between condiments, all of which were high-sugar foods, and composite Nutri-score; lunches containing condiments had a composite Nutri-score of D versus C in those without (Mann Whitney *U* = 397.00, *p* = 0.004). While other correlations were observed between composite Nutri-score and vegetables (Mann Whitney *U* = 369.50, *p* = 0.029), refined grains (Mann Whitney *U* = 326.50, *p* = 0.004), MFE (Mann Whitney *U* = 462.50, *p* < 0.001), dairy (Mann Whitney *U* = 545.00, *p* = 0.014) and snack foods (Mann Whitney *U* = 524.00, *p* = 0.002), calculations did not generate alternate Nutri-scores.

This study can be viewed positively in terms of feasibility, acceptability and fidelity. It was straightforward to implement and is reproducible. Children and parents were motivated to partake in the study, demonstrated by high recruitment and low refusal rates. Communication of the study scope and implications, via in-person presentation, would increase its acceptability. Expanding variables to include height and weight, activity levels and parental education level would add a deeper layer to the dataset but may hinder acceptability.

### 4.1 More colour variety equates to a healthier diet

The findings of this study are not consistent with previous studies that showed diet with colour variety was healthier. In a student population, prompts to eat colourful meals increased the proportion of healthy foods consumed [12]. Colourful meals were also ranked first for feasibility among this group [12]. The importance of visual cues, particularly food colour, on food choices and consumption has been previously highlighted [37,38]. Increased perceived meal colour variety has been associated with a healthier eating pattern [11,12].

Colours can arise due to phytochemical concentrations which enhance dietary healthfulness through interactions with essential nutrients [35]. Although no optimal ratio of colour has been identified, it is postulated that including a variety of colour in one's diet could result in better overall health [36].

The finding that the more colourful one's plate, the more pleasing it is to eat across age groups, geographical locations and cultures, is promising [41,42]. Children have been observed to opt for greater colour variety than their parents [41]. This indicates that meal presentation is of consequence in picky eaters and should be incorporated into eating guidelines.

### 4.2 Limitations

Due to COVID-19 restrictions imposed, it was not possible to better power the study. Our sample size is unlikely to find a significant relationship between variables, however strong associations may appear. Non-representativeness of the sample cannot not be ruled out. These factors might mean that the results may not be generalisable to the Irish population.

Nutri-scores were calculated by 100g, rather than based on portion size. This is important regarding the presence of foods, such as butter, where a small serving will vastly reduce the nutritional value of the lunchbox irrespective of what else it contains. This necessitates particular attention to ensure accuracy in the reporting of future work. An alternate method might involve weighing the contribution of individual foods to achieve increased accuracy. While weighing food requires additional time and resources, it would offer the added benefit of differentiating lunches containing a single combined food item, for example one sandwich versus multiple, which has implications in terms of caloric and nutrient intake.

## 5 Conclusion

No association was found between food colour variety and nutritional content in this small sample. Vegetable consumption was relatively low and refined grains were frequently observed. However, lunchboxes containing fruit and vegetables tended to be more colourful. Nutritional scores were poorer in younger children.

We suggest a means of feasibly adjusting dietary guidelines from reference range-guided to more easily-implementable recommendations, such as adding a colourful fruit or vegetable to a lunchbox or school-based encouragement to increase variety colours consumed each week in fruit and vegetables. This aligns with current healthy eating initiatives and could lead to improved nutritional intake in school-going children. Better-powered studies in a range of school settings are required to be able to make definitive decisions.

## Declaration of competing interest

The authors have no conflicts of interest to declare.
