# Supplementary material for: Does colour variety accurately quantify nutritional value in children's lunchboxes? A pilot study
Source: Public Health Pract (Oxf). 2023 Jan 31;5:100363. doi: 10.1016/j.puhip.2023.100363 (PMC9958369; doi:10.1016/j.puhip.2023.100363)
Supplement: Multimedia component 1 [file mmc1.docx]

Supplementary figure 6. Frequency of number of colours observed and composite Nutri-score per lunchbox by sex.

| 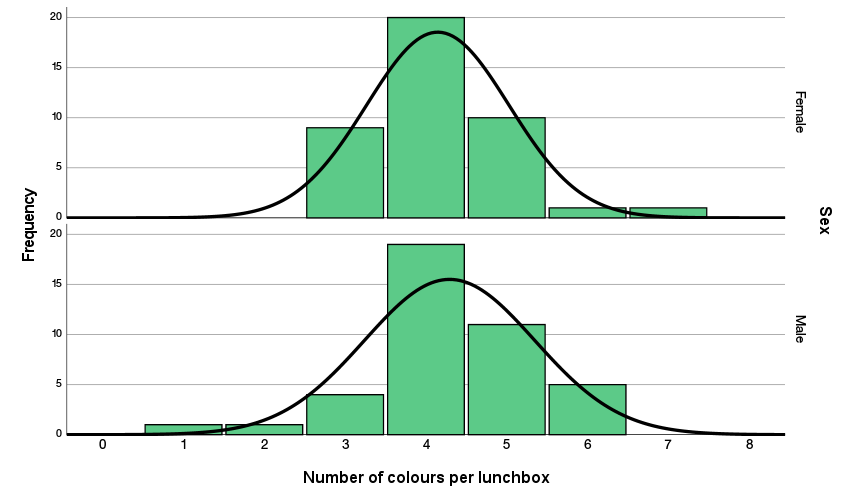 | 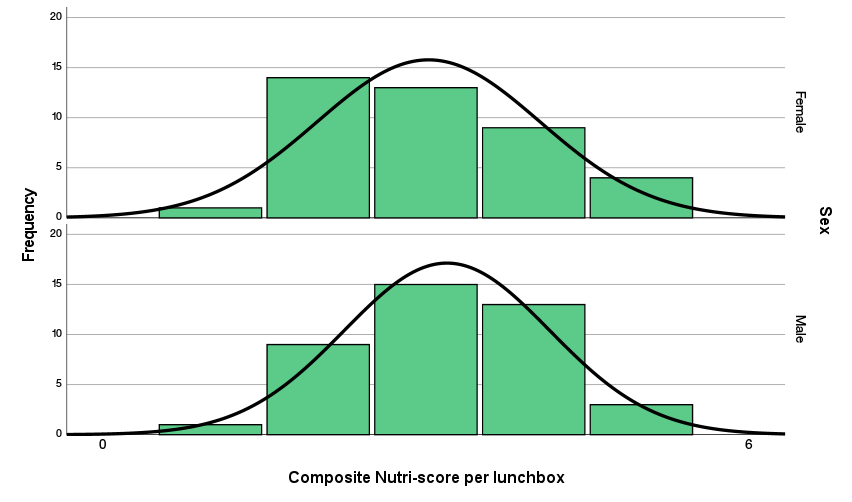 |
| --- | --- |
