# Supplementary material for: Does colour variety accurately quantify nutritional value in children's lunchboxes? A pilot study
Source: Public Health Pract (Oxf). 2023 Jan 31;5:100363. doi: 10.1016/j.puhip.2023.100363 (PMC9958369; doi:10.1016/j.puhip.2023.100363)
Supplement: Multimedia component 2 [file mmc2.docx]

# Supplementary figures and tables

Supplementary figure 1. Information leaflet and consent form for children.

My name is Dr Danielle Courtney and I am doing some research on colours in food. I need your help.

I am trying to find out if the more colourful your food is the more healthy it is. Can you help me? With your permission, I will take a photograph of your lunchbox one day in school. By looking inside your lunchbox, I will learn about what food you like to eat and about the colours of these foods.

Your name will not mentioned in the work. I will only need to take down your age and gender. These numbers will help to give a better understanding of whether food colour is important and how.

You do not have to take part in the project but it might be fun!

**Would you like to take part? Circle your answer.**


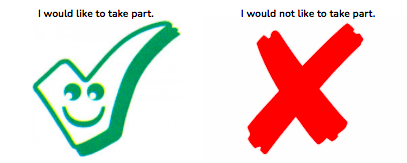


**Signature of the student: _____________________________________**

Supplementary figure 2. Information leaflet and consent form for parents.

**Does colour variety accurately represent nutritional value in children’s lunchboxes?**

Dear Parent / Guardian,

My name is Dr Danielle Courtney. I am currently undertaking a Master in Public Health in University College Cork. As part of the programme, I will complete research during the school year where I will investigate whether colour variety in children’s lunchboxes gives an indication of nutritional value. To do this successfully, I am seeking your support, as well as that of your child, to find ways of making it easier for parents and guardians to make lunchboxes as healthy as possible with limited time. My hope is that I can create a framework for use by parents and guardians in creating healthy lunches.

My research question is ‘Does colour variety accurately represent nutritional value in children’s lunchboxes?’ In approximately one month’s time, I will ask Principal XX and your child’s class teacher, Ms XX, to take photographs of your child’s lunchbox contents. The photographs of your child’s lunch will be taken of food only. If there is anything in the lunchbox to indicate the identity of your child, the food will be removed from the lunchbox and photographed separately. I will count the colours in the lunchbox in a bid to give a colour value to the lunchbox, a rainbow value. As well as photographs, I will ask Principal XX or Ms XX to record your child’s age on that day and their sex; these measurements will serve to indicate whether other factors have an impact on the colour of your child’s lunchbox contents. I require no sensitive information be gathered so your child will not be identifiable.

I hope that the results of this project can inform the government on how best to advise schools and parents on healthy eating and health promotion. You are welcome to receive feedback on its completion. If your child would like to withdraw their interest at any time, they are welcome to do so. I would like to invite them to participate in the project and direct any questions my way.

All participants' privacy and anonymity will be ensured at all times. You can contact me at any time to discuss this further at [120111290@umail.ucc.ie](mailto:120111290@umail.ucc.ie).

Thank you for your support.

Kind regards,

Dr Danielle Courtney

MCRN 406443

Supplementary table 3. Observed food in their assigned food groups for the purposes of analysis.

| **Food groups** | **Subgroups** | **Observed foods** |
| --- | --- | --- |
| **Fruit** | Whole fruit  Fruit juice  Mixed foods | Pineapple, banana, apple (red, green), grapes (red, green, black), melon, mandarin orange, peach, pear, strawberry, blueberry, blackberry, tomato (cherry, regular), bell pepper (yellow, red, green), cucumber  None observed  Innocent fruit smoothie |
| **Vegetables (including légumes)** | Dark green vegetables | None observed |
|  | Red and orange vegetables | Carrot |
|  | Beans and peas* | Green beans |
|  | Starchy vegetables | None observed |
|  | Other vegetables | Iceberg lettuce |
| **Grains** | Whole grains | Whole wheat bread |
|  | Refined grains | White bread, breadsticks, regular pasta, pretzels (soft-baked, dry), crackers (plain, cheese puff), croissant, pain au chocolat, Belgian waffle |
| **Protein foods** | Meat, fish & eggs | Chicken (plain, breaded), Pepperami meat stick, sliced ham |
|  | Nuts | Peanuts (ready salted and roasted), almonds (dry), walnuts (dry) |
| **Dairy products** | Milk and Yoghurt  Cheese | Natural yoghurt, yoghurt drinks, Müller corner yoghurt, Babybel cheese, Dairylea Dunkers, Cheesestrings, chocolate yoghurt, strawberry, Frube yoghurt, Petit filous yoghurt  Cheddar cheese |
| **Snack foods** | Rice cakes  Cereal and other breakfast bars  Biscuits  Sweets & chocolates  Other sweet snacks  Salty snack foods | Plain and chocolate  Cereal bars (store-branded, Nature Valley bar, Belvita), flapjacks (Flahavans)  Plain biscuit (Liga, Rich Tea, Tuc and other plain), wafer biscuits, Rice Krispie squares, Oreo biscuit  Hershey’s chocolate bar, Cadbury chocolate buttons, Twix bar, yellow Snack bar, marshmallows, sponge cake, pancakes  Raisins  Waffles crisps |
| **Fats & oils** | - | Butter |
| **Condiments** | - | Chocolate spread, jam (strawberry, raspberry), tomato sauce |
| *Beans and peas were considered as part of the Vegetables group or Protein group because they include important nutrients from both. [(38)](https://www.zotero.org/google-docs/?Bl96oe) | | |

Supplementary figure 4(a). STROBE checklist of items that should be included in reports of observational studies. [(20)](https://www.zotero.org/google-docs/?gf16E6)


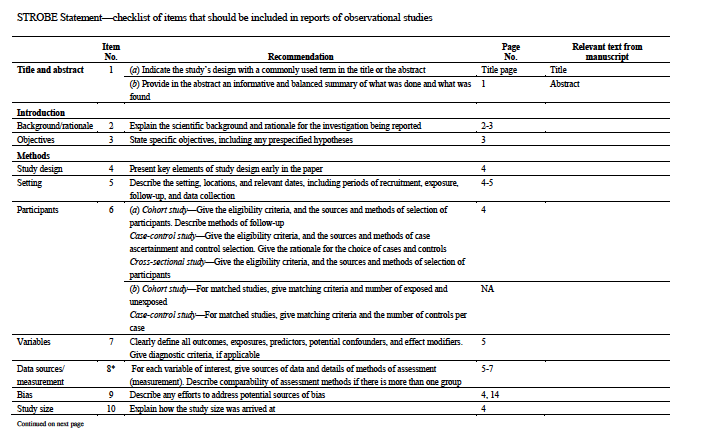


Supplementary figure 4(b). STROBE checklist of items that should be included in reports of observational studies. [(20)](https://www.zotero.org/google-docs/?gf16E6)


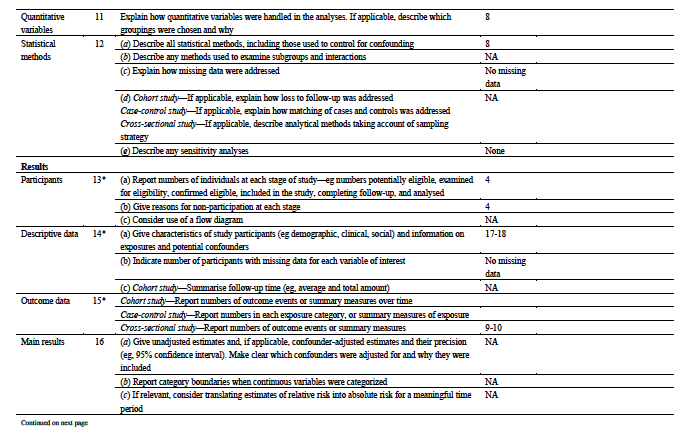


Supplementary figure 4(c). STROBE checklist of items that should be included in reports of observational studies. [(20)](https://www.zotero.org/google-docs/?gf16E6)


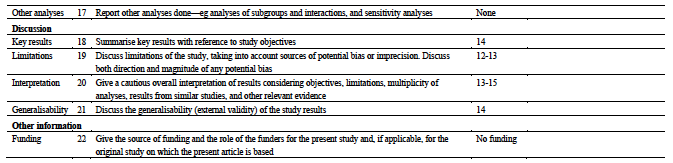


Supplementary table 5(a). Colour chart of total number of food items by food group and by colour.

| **Food groups** | **Colour chart** | | | | | | | | | | | | | **Number of food items observed**  (by food group) |
| --- | --- | --- | --- | --- | --- | --- | --- | --- | --- | --- | --- | --- | --- | --- |
|  | White | Yellow | Golden | Brown | Choc brown | Dark brown | Pink | Red | Orange | Green | Blue | Black | Purple |  |
| Fruit | - | 30 | - | - | - | - | - | 51 | 12 | 23 | 2 | 5 | 1 | 124 |
| Vegetable | - | - | - | - | - | - | - | - | 3 | 10 | - | - | - | 13 |
| Whole grains | - | - | 3 | 14 | - | - | - | - | - | - | - | - | - | 17 |
| Refined grains | 55 | 8 | 23 | - | - | - | - | - | - | - | - | - | - | 84 |
| Meat, fish and eggs | 4 | - | 1 | 1 | - | - | 28 | - | - | - | - | - | - | 34 |
| Nuts | - | - | 2 | 2 | - | - | - | - | - | - | - | - | - | 4 |
| Dairy | 25 | 4 | - | - | 1 | - | 6 | - | 25 | - | - | - | - | 61 |
| Snack foods | 9 | 9 | 23 | - | 10 | 4 | - | - | - | - | - | - | - | 55 |
| Fats and oils | - | 22 | - | - | - | - | - | - | - | - | - | - | - | 22 |
| Condiments | - | - | - | - | - | - | - | 9 | - | - | - | - | - | 9 |
| **Number of food items observed**  (by colour) | 93 | 73 | 52 | 17 | 11 | 4 | 34 | 60 | 40 | 33 | 2 | 5 | 1 | 425 |

Supplementary table 5(b). Colour chart of composite Nutri-score by food group and by colour.

| **Food groups** | **Colour chart** | | | | | | | | | | | | | **Composite**  **Nutri-score** (by food group) |
| --- | --- | --- | --- | --- | --- | --- | --- | --- | --- | --- | --- | --- | --- | --- |
|  | White | Yellow | Golden | Brown | Choc brown | Dark brown | Pink | Red | Orange | Green | Blue | Black | Purple |  |
| Fruit | - | B | - | - | - | - | - | A | C | A | A | B | B | B |
| Vegetable | - | - | - | - | - | - | - | - | A | A | - | - | - | A |
| Whole grains | - | - | A | A | - | - | - | - | - | - | - | - | - | A |
| Refined grains | B | A | C | - | - | - | - | - | - | - | - | - | - | B |
| Meat, fish and eggs | B | - | B | E | - | - | C | - | - | - | - | - | - | C |
| Nuts | - | - | A | C | - | - | - | - | - | - | - | - | - | B |
| Dairy | B | D | - | - | D | - | B | - | C | - | - | - | - | C |
| Fats and oils | D | D | C | - | E | D | - | - | - | - | - | - | - | D |
| Snack foods | - | D | - | - | - | - | - | - | - | - | - | - | - | D |
| Condiments | - | - | - | - | - | - | - | D | - | - | - | - | - | D |
| **Composite**  **Nutri-score**  (by colour) | B | C | B | C | D | D | C | B | B | A | A | B | B |  |

Supplementary figure 6. Frequency of number of colours observed and composite Nutri-score per lunchbox by sex.

| 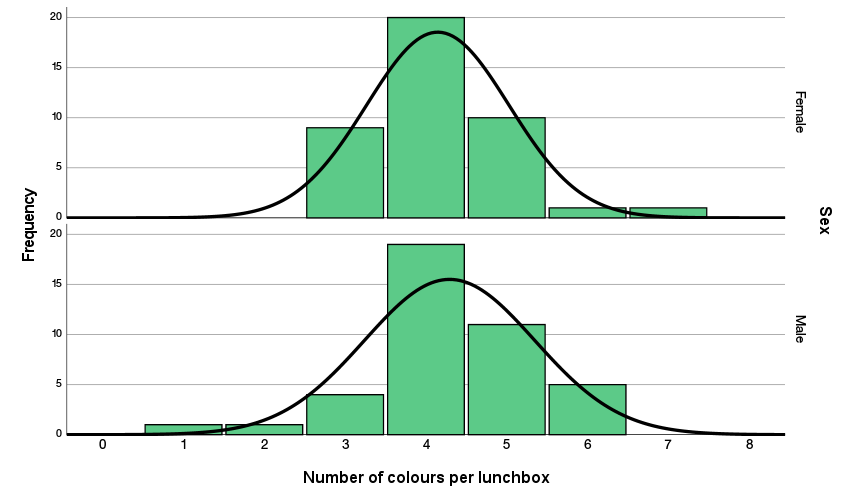 | 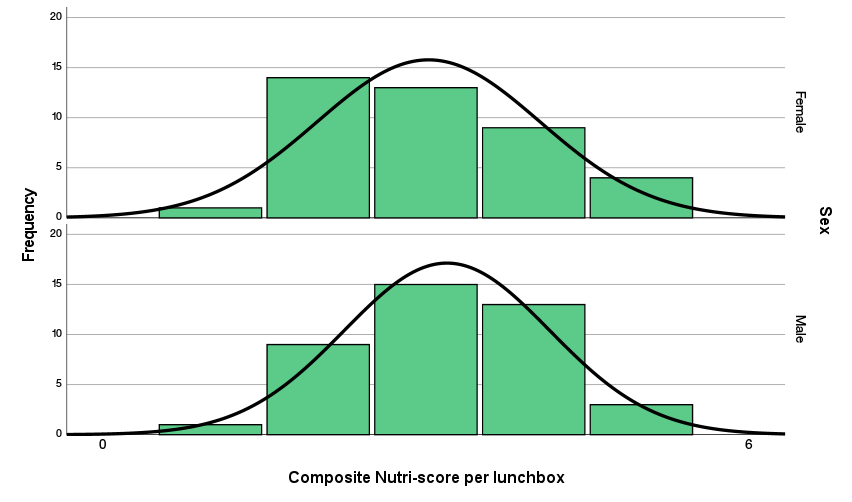 |
| --- | --- |

Supplementary figure 7. Frequency of number of colours observed and composite Nutri-score per lunchbox by age.

| 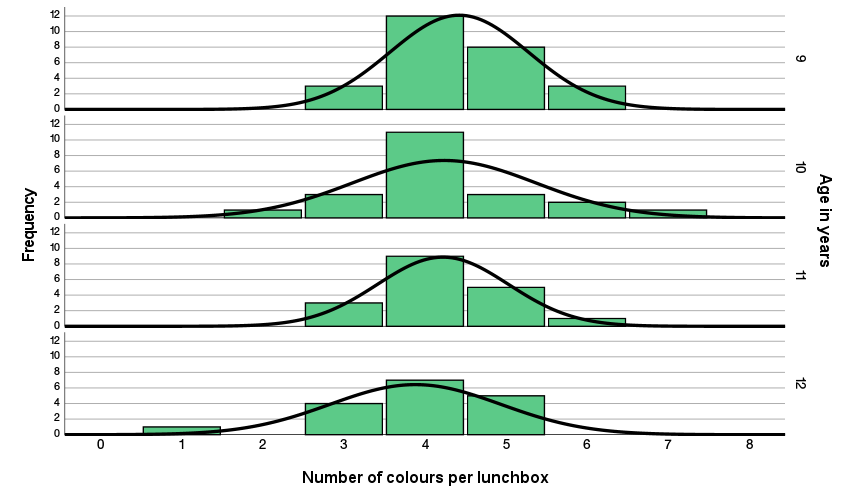 | 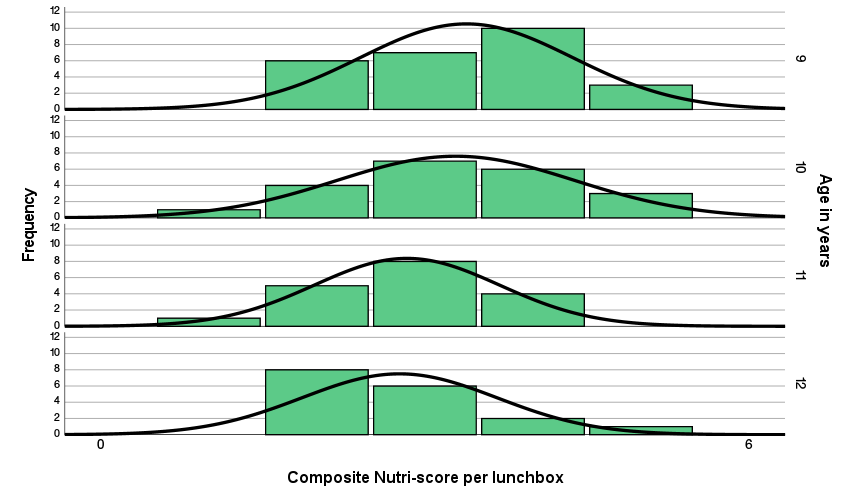 |
| --- | --- |

Supplementary table 8. Calculation of the reliability of observer ratings (*n*=11) of colours for the purposes of coding observed food items using Intra-Class Correlation.

|  | **Intraclass correlation^b^** | **95% confidence interval** | | **F test with true value 0** | | | |
| --- | --- | --- | --- | --- | --- | --- | --- |
|  |  | Lower bound | Upper bound | Value | df1 | df2 | Sig |
| **Single measures** | .838**^a^** | .794 | .879 | 60.134 | 90 | 900 | .000 |
| **Average measures** | .983**^c^** | .977 | .988 | 60.134 | 90 | 900 | .000 |

Two-way mixed effects model where people effects are random and measures effects are fixed.

**^a^** The estimator is the same, whether the interaction effect is present or not.

**^b^** Type A intraclass correlation coefficients using an absolute agreement definition.

**^c^** This estimate is computed assuming the interaction effect is absent, because it is not estimable otherwise.

# 
